# Clinical Management of Malignant Glaucoma

**DOI:** 10.1155/2015/283707

**Published:** 2015-12-24

**Authors:** Julie Foreman-Larkin, Peter A. Netland, Sarwat Salim

**Affiliations:** ^1^Leoni Eye Clinic, 203 Rue Louis XIV, Lafayette, LA 70508, USA; ^2^University of Virginia School of Medicine, Charlottesville, VA 22908, USA; ^3^Medical College of Wisconsin, 925 N 87th Street, Milwaukee, WI 53226, USA

## Abstract

Malignant glaucoma remains one of the most challenging complications of ocular surgery. Although it has been reported to occur spontaneously or after any ophthalmic procedure, it is most commonly encountered after glaucoma surgery in eyes with prior chronic angle closure. The clinical diagnosis is made in the setting of a patent peripheral iridotomy and axial flattening of the anterior chamber. Intraocular pressure is usually elevated, but it may be normal in some cases. Although the exact etiology of this condition is not fully understood, several mechanisms have been proposed and it is thought to result from posterior misdirection of aqueous humor into or behind the vitreous. This review discusses pathophysiology, differential diagnosis, imaging modalities, and current treatment strategies for this rare form of secondary glaucoma.

## 1. Introduction

Malignant glaucoma, initially described by von Graefe [1] in 1869, refers to a uniform shallowing or flattening of both the central and peripheral anterior chambers in an eye with normal to elevated intraocular pressure (IOP) despite one or more patent iridotomies. It was named “malignant” glaucoma because of its progressive course and poor response to conventional glaucoma therapy. This entity has been described by various terms, such as ciliary block glaucoma, aqueous misdirection, ciliolenticular glaucoma, and ciliovitreal block glaucoma, based on the possible mechanisms by which this constellation of clinical findings can be produced.

Although the exact etiology of this disease is not yet fully understood, it is believed to evolve from posterior misdirection of aqueous humor into or behind the vitreous. The resultant pressure differential between the posterior and anterior chambers causes an anterior displacement of the lens-iris diaphragm, anterior chamber shallowing or flattening, and secondary angle closure glaucoma. This review will focus on theories of mechanism and treatment strategies for this rare form of secondary glaucoma.

## 2. Epidemiology and Clinical Presentation

Malignant glaucoma is usually seen after incisional surgery, particularly glaucoma surgery in eyes with prior angle closure with a reported incidence of 2–4% [2]. However, it may also occur after laser surgery or any other intraocular surgery. It has been described after cataract surgery (with or without implant) [3–5], scleral buckle, pars plana vitrectomy, laser capsulotomy [6, 7], laser cyclophotocoagulation [8], laser iridotomy [9], and scleral flap suture lysis [10] and with the use of miotics [11, 12]. It has even been found to occur spontaneously in previously unoperated eyes [11, 13].

Malignant glaucoma has been documented postoperatively as soon as postoperative day one to as late as several years following intraocular surgery [14]. It has been associated with central retinal vein occlusions [15], inflammation, trauma, retinopathy of prematurity [16], intravitreal triamcinolone injection [17],* Aspergillus flavus* intraocular infection [18], and large intraocular lens [3]. Malignant glaucoma may be seen in phakic, aphakic, or pseudophakic eyes. It occurs more frequently in Asian eyes, probably due to their short axial length and predisposition to narrow anterior chamber angles [19]. In one report, the average age for patients with malignant glaucoma was 70 years with a female to male ratio of 11 : 3 [20].

On presentation, patients often complain of a red, painful eye with decreased vision, similar to symptoms reported with pupillary block glaucoma. Some may have associated headache with nausea and vomiting, depending on the level of IOP. Clinical examination shows axial flattening of the anterior chamber with anterior displacement of the lens, intraocular implant, or vitreous face, depending on the lenticular status of the eye in the presence of elevated IOP. Clear areas that represent entrapped aqueous fluid may be seen behind the posterior capsule or within the anterior vitreous [21]. Presence or absence of a patent iridotomy/iridectomy should be carefully noted to confirm this diagnosis and rule out other disease entities.

## 3. Pathophysiology

No theory has established a single cause of malignant glaucoma. Shaffer postulated that the forward shift of the lens-iris diaphragm in these patients is probably secondary to accumulation of aqueous humor behind a posterior vitreous detachment [22]. Later, the presence of aqueous pockets within the vitreous was shown by ultrasonographic studies conducted by Buschmann and Linnert [23]. Epstein [24] further elaborated on posterior diversion of aqueous at high IOP levels in his experiments on aged enucleated human eyes. Because of decreased permeability of the vitreous, the aqueous becomes trapped, causing a subsequent rise in intraocular pressure with secondary axial shallowing of the anterior chamber.

The alternative names of ciliolenticular block and ciliovitreal block are derived from the findings that the anterior rotation of the ciliary processes against the lens equator in phakic eyes or the anterior hyaloid face in aphakic eyes is responsible for lack of forward movement of the aqueous humor from the posterior chamber to the anterior chamber, causing a pressure differential in the two compartments [25, 26]. The anterior displacement of ciliary processes has been confirmed with ultrasound biomicroscopic studies, which have also shown the presence of shallow supraciliary detachments in these eyes, which may not be evident on routine B-scan imaging [19].

In 1972, Levene [27] hypothesized that increased IOP was a result of direct lens block, and the buildup of aqueous within the posterior cavity was a secondary feature. Lippas [25] along with others proposed ciliary spasm as the initiating event for anterior displacement of lens-iris diaphragm as a result of surgery, miotics, inflammation, or other causes.

It has also been suggested that anterior hyaloid obstruction may contribute as one of the underlying mechanisms. Quigley et al. [27, 28] hypothesized that choroidal expansion decreases the eye's ability to transmit aqueous freely across the vitreous. As vitreous compression increases with its displacement against the ciliary body, lens, or iris, the available area for fluid transport across the hyaloid membrane is diminished with reduced fluid conductivity, thereby prolonging the vicious cycle.

The consensus is that malignant glaucoma is a multifactorial disease in which more than one of the aforementioned mechanisms may play a role in its pathogenesis.

## 4. Differential Diagnosis

Pupillary block glaucoma should be considered in a patient with elevated IOP and flattening of the anterior chamber. However, the presence of a patent iridotomy/iridectomy helps rule out this entity. Unlike malignant glaucoma that produces uniform flattening of the anterior chamber, pupillary block glaucoma presents with iris bombe and shallow to flat peripheral anterior chamber but with moderate depth of the central anterior chamber. If the patency of an iridotomy is in question, a second iridotomy should be performed with an argon or neodymium:yttrium-aluminum-garnet (Nd:YAG) laser.

Choroidal detachments are common after glaucoma filtration surgery and may be confused with malignant glaucoma because of a shallow or flat anterior chamber depth. However, eyes with choroidal detachments are typically hypotonous. In some cases, IOP measurements may not be accurate in the setting of a flat anterior chamber, thereby making the distinction between the two conditions difficult. Choroidal effusions are usually light brown elevations and most resolve spontaneously. If the view to the fundus is impaired, these may be diagnosed with ultrasonography.

Suprachoroidal hemorrhage, which usually occurs hours or days after intraocular surgery and is often preceded by hypotony, should also be excluded by both clinical exam and ultrasound evaluation. The patient usually complains of severe throbbing eye pain. These eyes are typically more inflamed when compared to those with serous choroidal detachments. Clinically, choroidal elevations are present with a flat anterior chamber and elevated IOP. Ultrasound evaluation will reveal dome-shaped elevated choroidal hemorrhages with little to no movement on dynamic B-scan.

Additionally, in every patient after glaucoma filtration surgery, the possibility of a wound leak or overfiltration should be eliminated by careful examination as a possible etiology for a shallow or flat anterior chamber. Both of these conditions will have normal to low IOP.

## 5. Imaging Studies

**Figure 1:**
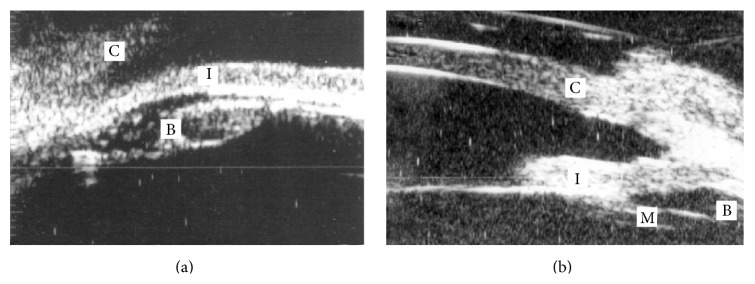
Ultrasound biomicroscopy (UBM) of malignant glaucoma. (a) The patient with a history of angle closure glaucoma and a patent laser iridotomy presented after glaucoma filtration surgery with elevated intraocular pressure. UBM showed shallow anterior chamber and anterior rotation of the ciliary body. (b) After treatment with cycloplegic medication and topical steroids, the anterior chamber deepened and the ciliary body returned to normal position. C, cornea; I, iris; B, ciliary body; and M, hyaloid membrane (reprinted by permission from [51]).

Ultrasound biomicroscopy (UBM) aids in both diagnosis and monitoring therapeutic response in eyes with malignant glaucoma. Park and colleagues reported malignant glaucoma in a pseudophakic eye, where UBM demonstrated an anterior rotation of the ciliary body and forward displacement of the lens haptic with apposition to the iris root [29]. Tello et al. [30] used UBM pre- and posttreatment in a pseudophakic eye with malignant glaucoma. Anterior rotation of the ciliary body and shallow anterior chamber depth were normalized after the anterior hyaloid face was disrupted with Nd:YAG laser. Anterior rotation of the ciliary body and shallow anterior depth were observed by UBM in 2 Asian patients with malignant glaucoma, controlled in both cases with cycloplegic medications (Figure 1) [31].

B-scan facilitates ruling out other causes of shallow or flat anterior chamber, such as suprachoroidal hemorrhage or choroidal effusions.

Optical coherence tomography (OCT) may be used as a noninvasive technique for monitoring anterior chamber narrowing in affected eyes. Wirbelaur et al. [32] used noncontact slit lamp adapted OCT to study the anterior chamber structures in eyes with malignant glaucoma after trabeculectomy. They reported both qualitative and quantitative marked shallowing of the anterior chamber depth during the acute presentation and resolution of these findings after pars plana vitrectomy and deepening of the anterior chamber with viscoelastic agents.

## 6. Management

### 6.1. Medical Management

Medical management is usually tried for approximately 3 to 5 days before surgical intervention is attempted, depending on the clinical findings. First-line agents include mydriatic-cycloplegic agents and aqueous suppressants. In 1962, Chandler and Grant [33] popularized the cycloplegic therapy in treating malignant glaucoma. Cycloplegics tighten the lens zonules by relaxing the ciliary muscle, pulling the lens-iris diaphragm posteriorly, and alleviating the ciliary block. The aqueous suppressants decrease the posterior pooling of the aqueous humor by reducing its production. The use of hyperosmotics was supported by Daniele and Diotallevi [34] when they reported success with intravenous urea. This treatment was further endorsed by Weiss et al. [26] who used intravenous mannitol. Hyperosmotic agents dehydrate the vitreous, allowing posterior movement of the lens-iris diaphragm with expansion of anterior chamber space.

Whether cycloplegics should be used alone or in combination with hyperosmotics in the initial management of malignant glaucoma was studied by Chandler and colleagues [35]. They reported greater success with the combination therapy: resolution in 9 out of 19 cases compared to cycloplegic therapy alone where 5 out of 11 eyes showed reversal. The decision to use combination therapy is ultimately a clinical one based on the exam, degree of pressure elevation, and the extent of glaucomatous damage at the time of presentation.

Simmons reported that approximately 50% of patients with malignant glaucoma respond to medical treatment alone and further refined Chandler's course of treatment [36]. A typical regimen includes atropine 1% four times daily (QID) to relax the ciliary muscle, phenylephrine 10% QID, an *α*1 adrenergic agonist, to stimulate the iris dilator muscle, hyperosmotics, either glycerol 50% orally (1 mL per pound body weight) daily or mannitol (2 g per kg body weight) orally daily or twice daily, to decrease vitreous volume, and topical or systemic aqueous suppressants to decrease aqueous pooling posteriorly. If oral agents such as acetazolamide are to be used, electrolytes should be monitored frequently, especially potassium levels.

As described earlier, patients should be maintained on this regimen for approximately 3 to 5 days to monitor clinical improvement. Confounding factors in the patient's clinical situation, such as corneal decompensation from lens apposition against the corneal endothelium, may require more rapid clinical intervention. If the patient responds to aggressive medical therapy, the treatment can be gradually tapered by discontinuing the hyperosmotics initially and then the aqueous suppressants and finally phenylephrine and atropine. Studies have shown that patients may have to be maintained on cycloplegic agents indefinitely because of the high risk of recurrence with the cessation of these agents [37].

### 6.2. Surgical Management

**Figure 2:**
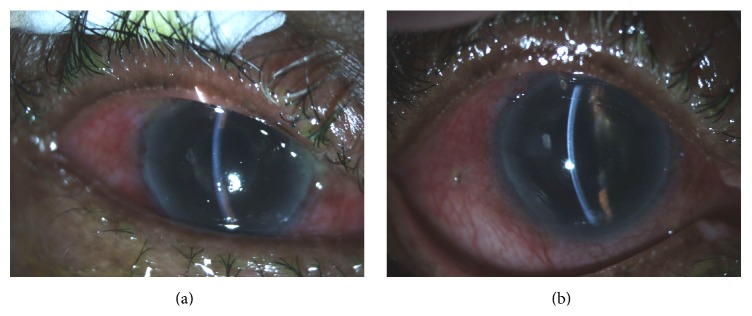
Malignant glaucoma treatment. (a) The patient presented after glaucoma filtration surgery with a shallow chamber and markedly elevated intraocular pressure, not responding to initial treatment with cycloplegia and laser. (b) After pars plana vitrectomy, the anterior chamber was deep and the intraocular pressure was normalized.

In refractory cases, laser or surgical intervention is usually indicated. Argon laser treatment may shrink the ciliary processes through a patent iridotomy/iridectomy; Nd:YAG laser may be used to rupture the posterior capsule and anterior hyaloid membrane. Peripheral iridotomy should be performed initially to exclude pupillary block mechanism or if there is a question of the patency of a previously existing iridotomy. Pars plana vitrectomy is effective for this condition (Figure 2).

Nd:YAG laser capsulotomy and hyaloidotomy should be considered in pseudophakic and aphakic eyes. The aim of this procedure is to disrupt the anterior hyaloid and eliminate it as a fluid barrier to allow movement of fluid between the posterior and anterior segments of the eye. Several series have reported success with this procedure in eyes refractory to medical therapy [21, 38, 39]. Little and Hitchings [21] suggested placing the posterior capsulotomy peripheral to the lens to avoid the ensuing blockage of aqueous flow. Nd:YAG laser should be avoided in phakic patients because of the risk of damaging the lens.

A previously made peripheral iridotomy/iridectomy may provide ample view for the Nd:YAG laser to be focused through the aperture behind the posterior capsule at the peripheral hyaloid. Frequently, a posterior capsulotomy is performed first with the assumption that the posterior capsule should be removed first in order to achieve adequate breaks in the hyaloid. Moderate deepening of the anterior chamber should be seen over the next 24 h if this procedure is effective in providing free flow of fluid between the posterior and anterior chambers.

If Nd:YAG laser capsulotomy and hyaloidotomy are ineffective, many physicians proceed to pars plana vitrectomy with surgical removal of the anterior hyaloid face. This approach was initially described by Chandler who used a large diameter needle (18 g) inserted through the pars plana to remove vitreous and trapped aqueous [40]. Several studies have reported success after a single vitrectomy [2, 41]. Harbour et al. [41] pointed out several advantages of vitrectomy over other surgical procedures: (1) the exact location of obstruction does not have to be identified, (2) the risk of recurrence is low because of removal of vitreous, and (3) visibility and safety are improved. In their study, 21 of 24 eyes showed improvement with initial vitrectomy; however, some cases were associated with severe postoperative complications [41].

For phakic eyes, some surgeons recommend lensectomy at the time of vitrectomy because of the increased incidence of postoperative cataract formation. Harbour et al. [41] also demonstrated that eyes undergoing vitrectomy without lensectomy had a lower success rate. Therefore, it was recommended that lens extraction should be considered in eyes where the anterior chamber did not deepen intraoperatively, in eyes with prominent corneal edema from lens-cornea apposition, and in eyes where dense cataracts were present at the time of surgery.

Debrouwere et al. [42] performed a retrospective review comparing the relapse rates of different therapeutic interventions. They found the combined technique of vitrectomy-iridectomy-zonulectomy (and phacoemulsification if the patient was phakic) to have the lowest relapse rate compared to vitrectomy or YAG capsulotomy with hyaloidotomy. In pseudophakic eyes, iridectomy-hyaloidotomy-zonulectomy combined with anterior vitrectomy were also associated with lower recurrence rates.

Other surgical techniques include posterior sclerotomy [43], anterior chamber reformation [25], transscleral cyclodiode laser photocoagulation [44], and vitreous puncture and aspiration [35]. However, these are less widely used. In the presence of peripheral anterior synechiae formation and fibrosis in the anterior segment, glaucoma drainage implant surgery or goniosynechialysis may be of value combined with pars plana vitrectomy [45].

## 7. Associated Features/Complications

Several sources have found an association of myopic shift ranging from −2.5 to −8 diopters because of anterior displacement of the lens-iris diaphragm during an attack of malignant glaucoma [46]. Sii and Shah [47] reported an extreme myopic shift of −8.0 diopters in a healthy hyperopic male with a history of chronic angle closure glaucoma requiring prior Nd:YAG laser peripheral iridotomies and trabeculectomy. The patient developed malignant glaucoma after laser suture lysis and was treated medically with successful reduction of IOP.

Failure of prior functioning trabeculectomy for chronic angle closure glaucoma may occur after vitrectomy for treatment of malignant glaucoma [2, 40, 48]. Azuara-Blanco et al. [49] reported two cases that ultimately required Baerveldt tube shunt placement through the pars plana for failed trabeculectomy after vitrectomy which had initially been performed for malignant glaucoma.

Cataract formation has been found in a higher percentage of patients undergoing vitrectomy for malignant glaucoma [2, 41]. Retinal detachment and serous choroidal detachments have also been reported after surgical intervention for malignant glaucoma [2].

## 8. Treatment of the Fellow Eye

Patients with a diagnosis of malignant glaucoma in the affected eye have an increased risk of malignant glaucoma in the fellow eye. Many physicians perform prophylactic iridotomy/iridectomy in the fellow eye if the drainage angle is found to be narrow or closed before any surgical intervention. Miotics should be avoided in these eyes, and aggressive cycloplegic therapy should be instituted after surgery. Prophylactic vitrectomy at the time of planned phacoemulsification can be considered, especially in the case of a patient who had severe aqueous misdirection in the fellow eye requiring vitrectomy [50].
